# A Real-World Study of Achievement Rate and Predictive Factors of Clinical and Deep Remission to Biologics in Patients with Severe Asthma

**DOI:** 10.3390/jcm12082900

**Published:** 2023-04-16

**Authors:** Keiji Oishi, Kazuki Hamada, Yoriyuki Murata, Kazuki Matsuda, Syuichiro Ohata, Yoshikazu Yamaji, Maki Asami-Noyama, Nobutaka Edakuni, Tomoyuki Kakugawa, Tsunahiko Hirano, Kazuto Matsunaga

**Affiliations:** 1 Department of Respiratory Medicine and Infectious Disease, Graduate School of Medicine, Yamaguchi University, Ube 755-8505, Japan; 2 Department of Pulmonology and Gerontology, Graduate School of Medicine, Yamaguchi University, Ube 755-8505, Japan

**Keywords:** biologics, clinical remission, deep remission, early introduction, severe asthma

## Abstract

Background: Recent advances in biologics have provided new insights into the clinical course of asthma, including disease modification, clinical remission (CR), and deep remission (DR). However, the extent to which biologics achieve CR and DR in severe asthma patients is poorly understood. Methods: To assess the achievement rate and predictors of CR and DR using long-term biologics, we retrospectively evaluated 54 severe asthma patients recently started on biologics. “CR” denotes the achievement of all three criteria: (1) absence of asthma symptoms, (2) no asthma exacerbations, and (3) no use of oral corticosteroids. DR denoted CR plus (4) normalized pulmonary function and (5) suppressed type 2 inflammation. Results: CR and DR achievement rates were 68.5% and 31.5%, respectively. Compared with the non-deep remission group, the DR group had higher adult-onset asthma rates (94.1% vs. 70.3%, *p* = 0.078), shorter asthma duration (5 vs. 19 years, *p* = 0.006), and higher FEV_1_ (91.5% vs. 71.5%, *p* < 0.001). There were no significant differences in the Asthma Control Questionnaire scores, exacerbation frequency, or type 2 inflammation at baseline between groups. Asthma duration combined with FEV_1_ can stratify the achievement rates of CR and DR. Conclusions: the early introduction of biologics in severe asthma patients may help achieve CR and DR.

## 1. Introduction

Asthma is a common, heterogeneous, and chronic inflammatory airway disease characterized by a history of respiratory symptoms, such as wheezing, shortness of breath, chest tightness, and cough. The type and intensity of asthma symptoms vary over time, together with variable expiratory airflow limitation [1]. Approximately 5–10% of asthma patients are diagnosed with severe asthma and continue to exhibit uncontrolled symptoms despite optimal management, including high-dose inhaled corticosteroids [2,3]. Patients with severe asthma are frequently treated with oral corticosteroids (OCS) [4]; however, long-term OCS treatment is associated with an increased risk of comorbidities and mortality [5,6].

An estimated 70–80% of patients with severe asthma display signs of type 2 (T2) inflammation [7,8], which is clinically defined as increased blood and airway eosinophils, elevated fraction of exhaled nitric oxide (FeNO) levels, and elevated levels of serum immunoglobulin E (IgE). Recently, biologics targeting mediators of T2 inflammation have been shown to dramatically improve the management of severe asthma patients with T2 inflammation [9,10]. Biologics have provided new insights into the clinical course of asthma, including disease modification and remission.

Compared with asthma, chronic inflammatory diseases such as rheumatoid arthritis (RA) have a long history of biological therapy. The treatment goals for patients with RA are remission and sustained reduction in disease activity, which consist of a combination of various parameters. The discontinuation of biological therapy is generally considered in patients with RA who achieve deep remission [11]. Therefore, it is necessary to focus on deep remission as the next step in the management of severe asthma. Several expert groups have recently proposed definitions of clinical/complete remission and bio-free remission for severe asthma [12,13,14]. These definitions include various aspects of asthma, including symptoms, exacerbations, lung function, and airway inflammation. This includes suppressing T2 inflammation (immunological remission), normalizing pulmonary function by improving airway remodeling and obstruction (functional remission), maintaining symptom control, and preventing exacerbations under OCS-free conditions (clinical remission), ultimately leading to deep remission. However, the extent to which biologics achieve clinical and deep remission in patients with severe asthma remains poorly understood. This study aimed to assess the achievement rate and predictive factors of clinical and deep remission (clinical remission plus functional and immunological remissions) using long-term biologics in patients with severe asthma.

## 2. Materials and Methods

### 2.1. Study Design

We collected data from patients with uncontrolled severe asthma who initiated treatment with biologics at the Yamaguchi University Hospital between January 2016 and December 2021. All medical records were retrospectively reviewed. Severe asthma was defined as asthma that is uncontrolled despite adherence with optimized high-dose ICS plus long-acting beta 2-agonist (LABA) therapy and treatment of contributory factors or that worsens when high-dose treatment is decreased [1,2]. Uncontrolled asthma was defined as having at least one of the following: (1) poor symptom control (Asthma Control Questionnaire (ACQ) ≥ 1.5); (2) frequent severe exacerbations (two or more episodes of systemic corticosteroid use in the previous year); or (3) airflow limitation (forced expiratory volume in one second (FEV_1_) < 80% predicted) [2]. Based on the Japanese guidelines for adult asthma from the Japanese Society of Allergology (JSA), biologics should be prescribed for severe asthma patients with uncontrolled T2 inflammation who do not respond well to standard asthma treatments, such as high-dose ICS plus LABA [15]. T2-high was determined by the presence of atopy and/or FeNO ≥ 35 ppb and/or blood eosinophils ≥ 300/μL, while T2-low was defined by the absence of all these characteristics [15]. Our criteria for the use of biologics in severe asthma patients with uncontrolled T2-high are in accordance with the Japanese guidelines for adult asthma from the JSA. Patients who discontinued biological treatment within one year were excluded from the study. The choice of biologic was based on the judgment of each physician. The study protocol and amendments were approved by the Ethics Committee of Yamaguchi Medical University (Institutional Review Board number: 2021-039). The requirement for informed consent was waived by the ethics committee because no invasive procedures, interventions, or human samples were used in this retrospective study and anonymity was secured. This study complied with the Japanese Ethical Guidelines for Medical and Health Research Involving Human Subjects [16], which do not require informed consent from patients enrolled in studies that do not use human biological specimens. However, we provided participants with the choice to opt out of the study by announcing the study information on the bulletin boards in the hospital and on hospital websites.

The primary endpoint of this study was to evaluate the achievement rate of clinical and deep remission to biologics in patients with severe asthma. The secondary endpoint was to investigate the predictors of clinical and deep remission.

### 2.2. Study Assessments

Forced vital capacity (FVC) and FEV_1_ were measured using a dry rolling seal spirometer (CHESTAC-8800; Chest Co., Tokyo, Japan). The predictive values were estimated using the Japanese Respiratory Society prediction formula. FeNO was measured using a widely used electrochemical nitric oxide analyzer (NIOX VERO; Circassia Pharmaceuticals plc, Oxford, UK), as previously described [17]. Complete blood cell count, differential leukocyte count, and total serum IgE levels were examined. Specific IgE for common inhaled allergens, including house dust mites, *anthoxanthum odoratum*, *chamomile*, *ragweed*, *mugwort*, *Aspergillus*, *Candida*, dogs, and cats, was examined using the ImmunoCAP system (Pharmacia Diagnostics, Uppsala, Sweden). Positive specific IgE (>0.7 UA/mL) for at least one allergen was required to confirm the presence of atopy. The duration of asthma was defined as the period from the diagnosis of asthma to the initiation of biologic therapy. Obesity was defined as a body mass index greater than 25 kg/m^2^ [18]. The ACQ score, a validated tool for measuring asthma control [19,20], was used to assess asthma control in this study. According to the American Thoracic Society/European Respiratory Society statement, asthma exacerbation is defined as the worsening of asthma requiring the use of systemic corticosteroids or an increase from a stable maintenance dose for at least 3 days [20].

### 2.3. Definitions of Clinical and Deep Remissions

Clinical remission was defined as the absence of significant asthma symptoms (ACQ < 1.5) at the 1-year follow-up, no use of maintenance OCS at the 1-year follow-up, no use of burst OCS in the past 1 year, and no exacerbation during the 1-year period after the introduction of biologics. Functional remission was defined as having normalized pulmonary function (FEV_1_ ≥ 80% predicted) at the 1-year follow-up. Immunological remission was defined as suppressed T2 inflammation (blood eosinophils < 300 cells/μL and FeNO < 35 ppb) [15] at the 1-year follow-up. Deep remission was defined as meeting all these criteria.

### 2.4. Statistical Analysis

Data are presented as the median (interquartile range). Comparisons between groups were performed using the Mann–Whitney U test for non-normally distributed continuous variables or the chi-squared and Fisher’s exact tests for proportional data. The Wilcoxon signed-rank test was used to compare the paired data for each patient. The threshold value of each continuous variable was determined by the optimal cutoff value using a receiver operating characteristic (ROC) curve. The optimal cut-off values were obtained based on the highest Youden index values. Binary logistic regression analysis was performed to assess whether the variables were predictors of clinical and deep remission. Multivariate logistic regression analysis was not performed because the sample size was too small to obtain statistical power. Statistical significance was set at *p* < 0.05. Statistical analyses were performed using JMP Pro (version 16.0.0; SAS Institute Inc., Cary, NC, USA).

## 3. Results

### 3.1. Patient Characteristics at Baseline and after 1-Year Follow-Up of Biologics

Fifty-four patients were included in the study. None of the patients achieved clinical and/or functional remission at the baseline. The clinical characteristics of the study population at baseline are shown in the left panel of Table 1. The median age of the patients was 61 years. Approximately half of the patients were men, and forty percent were former smokers. Approximately 60% of the patients had allergic rhinitis, and 70% had chronic rhinosinusitis (CRS). All patients were prescribed high-dose ICS and LABA, and 37% required maintenance OCS therapy. The type of biologic used was generally uniform, with benralizumab being the most commonly used biologic.

The clinical characteristics of the biologics after the 1-year follow-up are shown in the right panel of Table 1. Blood eosinophil counts and FeNO significantly decreased 1 year after biologics, while the ACQ score and FEV_1_ % predicted significantly decreased and increased, respectively. Among patients requiring maintenance OCS therapy at baseline, maintenance OCS doses were significantly lower 1 year after biologics. Approximately two-thirds of patients discontinued maintenance OCS therapy.

### 3.2. Changes in the Proportion of Achievement of the Deep Remission Component after Initiating Biologics

**Figure 1:**
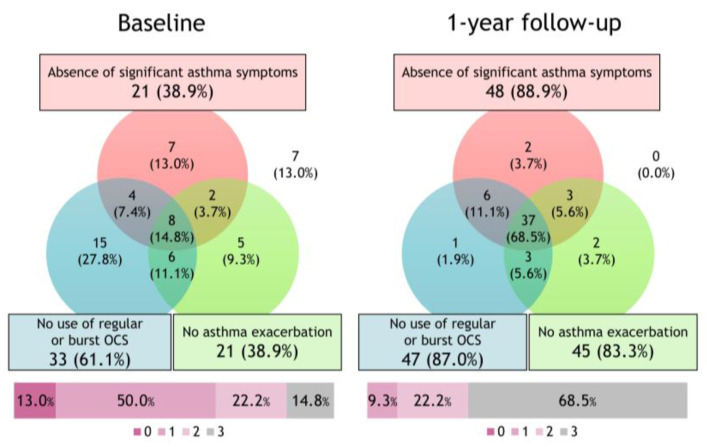
Changes in clinical remission subtypes after initiating biologics. The Venn diagram and percentage of patients achieving the three components of clinical remission before and after the initiation of biologics. With the initiation of biologics, the proportion of patients in clinical remission increased from 14.8% to 68.5%.

The Venn diagram and percentage of patients achieving the three components of clinical remission before and after the initiation of biologic therapy are illustrated in Figure 1. After the 1-year follow-up, the achievement rates for the absence of significant asthma symptoms, no use of OCS, and no asthma exacerbation were 88.9%, 83.3%, and 87.0%, respectively. With the initiation of biologics, the proportion of patients in clinical remission increased from 14.8% to 68.5%.

**Figure 2:**
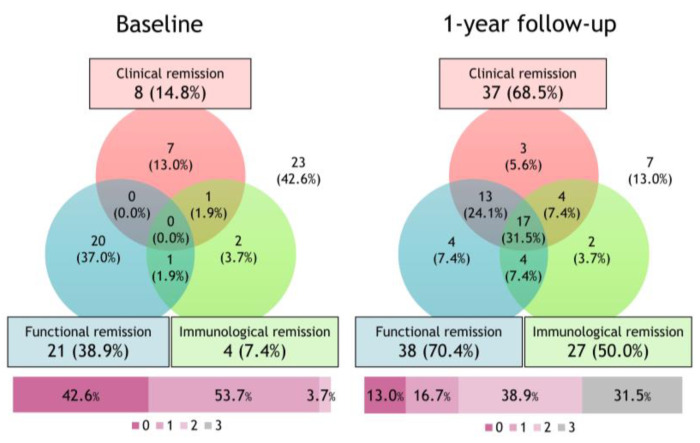
Changes in deep remission subtypes after initiating biologics. The Venn diagram and percentage of patients achieving the three components of deep remission before and after the initiation of biologics. With the initiation of biologics, the proportion of patients in deep remission increased from 0.0% to 31.5%.

Figure 2 shows the Venn diagram and percentage of patients achieving the three components of deep remission before and after the initiation of biologics. The proportion of patients who did not achieve one of the three remissions with biological treatment decreased from 42.6% to 13.0%. After the 1-year follow-up, the achievement rates of clinical, functional, and immunological remission were 68.5%, 70.4%, and 50.0%, respectively. Seventeen patients (31.5%) achieved deep remission.

### 3.3. Comparison of Patient Characteristics between the Clinical Remission and Non-Clinical Remission Groups

Characteristics of the clinical and non-clinical remission groups were compared (Table 2). The clinical remission group showed a significantly shorter duration of asthma, lower ACQ, and higher FEV_1_ % predicted. The proportions of never-smokers and patients with adult-onset asthma were higher in the deep remission group than in the non-deep remission group. There were no significant differences between the two groups with respect to age, T2 biomarkers, comorbidity, exacerbation history, or treatment medication, except for the use of maintenance OCS.

### 3.4. Comparison of Patient Characteristics between the Deep Remission and Non-Deep Remission Groups

The characteristics of the deep remission and non-deep remission groups were compared (Table 3). The deep remission group showed a significantly shorter duration of asthma, a higher FEV_1_ % predicted, and a higher rate of adult-onset asthma. There were no significant differences between the two groups with respect to age, T2 biomarkers, comorbidities, exacerbation history, ACQ, treatment medication, or the number of remission criteria achieved.

### 3.5. Predictors of Clinical and Deep Remission to Biologics

Duration of asthma and FEV_1_ % predicted showed significant differences between the deep remission and non-deep remission groups and were examined for their usefulness as predictors of clinical and deep remission in response to biologics. The optimal cutoff value for the duration of asthma was 5 years, and the area under the ROC curve (AUC) was 0.736. The sensitivity and specificity were 58.8% and 83.8%, respectively. The optimal cut-off value of FEV_1_ % predicted was 75, and AUC was 0.797. The sensitivity and specificity were 94.1% and 64.8%, respectively.

**Figure 3:**
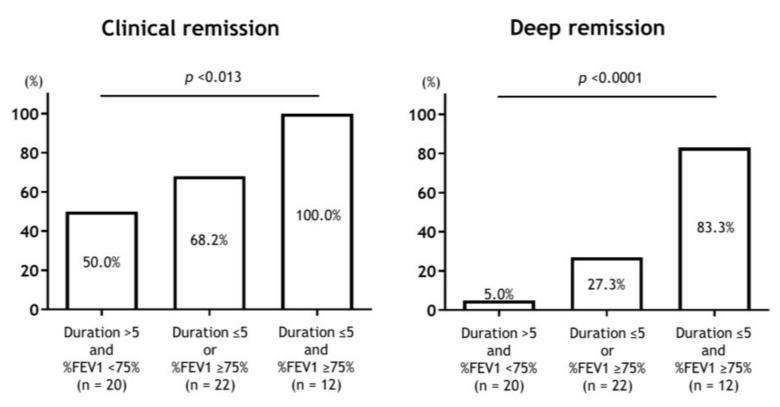
Achievement rates of clinical and deep remission according to the levels of asthma duration and airflow limitation. There was a significant difference in the achievement rate of clinical and deep remission according to the asthma duration and airflow limitation.

The results concerning the predictors of clinical and deep remission with biologics using logistic regression analysis are shown in Table 4 and Table 5. Asthma duration and FEV_1_ % predicted were predictors of clinical remission, with an odds ratio of 10.91 and 3.38, respectively. Asthma duration and FEV_1_ % predicted were predictors of deep remission, with odds ratios of 7.38 and 26.29, respectively. The achievement rates of clinical and deep remission stratified by asthma duration and FEV_1_ % predicted are shown in Figure 3. There was a significant difference in the achievement rates of clinical and deep remission according to the duration of asthma and airflow limitation.

## 4. Discussion

In this study, we focused on clinical and deep remissions by long-term biologics in patients with severe asthma. We found that the majority of patients achieved several composite remission components, with 68.5% achieving clinical remission and 31.5% achieving deep remission. We demonstrated that shorter asthma duration and higher %FEV_1_ may be associated with the possibility of achieving clinical and deep remission after initiating biologics.

In this real-world study with a 1-year response to biologics in severe asthma, the majority of patients achieved multiple composite remission components, with two-thirds achieving clinical remission and one-third achieving deep remission. Several studies have evaluated the incidence of the composite outcome of remission in patients with severe asthma treated with biologics. According to a recent post hoc study of LIBERTY ASTHMA QUEST, 20% of the participants who received dupilumab attained composite remission components at 12 months (no exacerbation, ACQ-5 < 1.5, and post-bronchodilator FEV_1_ ≥ 80%) [21]. Similarly, a post hoc analysis of SIROCCO/CALIMA of benralizumab showed that 15% of the patients treated with benralizumab achieved clinical remission (no exacerbations, no OCS use, ACQ-6 score ≤ 0.75, and pre-bronchodilator FEV_1_ increase ≤ 100 mL) at 12 months [22]. A post hoc analysis of the real-world REDES study, where the participants were treated with mepolizumab for 1 year demonstrated that 37% of patients achieved clinical remission with an Asthma Control Test score ≥ 20, while being OCS-free and without exacerbation [23]. A prospective real-world study of severe eosinophilic asthma treated with several anti-interleukin (IL)-5 biologics (mepolizumab, reslizumab, and benralizumab) for 2 years revealed that 14% of patients were super-responders who met all of the following criteria: no chronic OCS use, no OCS bursts in the past 3 months, ACQ score < 1.5, FEV_1_ ≥8 0% predicted, FeNO < 50 ppb, and complete control of comorbidities [24]. Although the definition of super responders was similar to that of deep remission, it differed slightly in terms of duration without OCS bursts (3 vs. 12 months) and FeNO (< 50 ppb vs. < 35 ppb). The achievement rate varied considerably among the studies, mainly owing to the clinical heterogeneity among the study populations, methods, definitions of remission, and assessments. However, the achievement rates of clinical and deep remission, as defined by our strict criteria, and the individual remission components in our study were comparable or superior to those reported in previous studies. Among them, the achievement rate of the three components of clinical remission was extremely high at more than 80%. These high efficacies may be related to the characteristics of Japanese patients with severe asthma, such as fewer obese patients and smokers than those in other countries. To the best of our knowledge, this is the first study to analyze the incidence of the composite outcome of remission in Japanese patients with severe asthma. Japanese data were used to evaluate individual remission components, such as exacerbation and OCS reduction. Several post hoc analyses of the Japanese subgroup in randomized controlled trials (RCTs) of biologics have revealed that the improvements observed in the Japanese cohort were more pronounced than those in the overall population [25,26].

Several studies have assessed factors associated with remission in adult patients with asthma. Remission was consistently associated with baseline variables, such as mild asthma, better lung function and asthma control, younger age, early onset asthma, shorter asthma duration, lower airway hyperresponsiveness (AHR), smoking cessation or never smoking, and fewer comorbidities [27]. Notably, while all of these studies examined spontaneous remission, few examined treatment-induced remission in patients with severe asthma. The current study, which targeted treatment-induced remission, demonstrated that several characteristics, such as shorter asthma duration and higher %FEV_1_, may be associated with the possibility of achieving clinical and deep remission after initiating biologics. Our results are in line with those of a previous study showing that higher FEV_1_ and shorter asthma duration were associated with super responders after 2 years of anti-IL-5 treatment [24]. This finding suggests that early initiation of biologics in severe asthma could modify the clinical course and prognosis. Biologics were initially used to treat severe end-stage RA. Subsequently, they were introduced at an earlier stage of disease management to modify disease progression, leading to lasting treatment-free remission in certain patients [28]. Early phenotyping and rapid introduction of biologics for patients with severe asthma may first control T2 inflammation and then reduce the exposure of the airway wall to various inflammatory mediators, improving AHR and airway remodeling, and finally halting disease progression [27,29]. Recently, a review of the stepwise approach and the concept of treatable traits has attracted attention as a new paradigm for asthma management [30,31]. Various treatment strategies should be discussed, and further studies are necessary to determine whether early biological interventions can lead to deep remission.

Compared to the non-clinical remission group, the clinical remission group had a lower baseline ACQ score and fewer patients with maintenance OCS use. This may be because ACQ values and maintenance OCS use were included in the definition of clinical remission. Adult-onset asthma was observed more frequently in the deep remission group than in the non-deep remission group. Because adult-onset asthma is a potential confounding factor for asthma duration, further studies are needed to determine whether this factor is predictive. Many RCTs and real-world studies have reported that T2 biomarkers, such as blood eosinophils and FeNO, are predictors of the response to each biological treatment [32]. T2 biomarker profiles were not significantly different between the two groups. This might be attributed to the fact that the study group completely composed of the T2-high subtype, and the majority of patients overlapped with all three T2 biomarkers. In addition, the sample size of our study was limited, and further studies are required to confirm our findings.

In contrast, deep remission was not achieved in two-thirds of patients. The achievement rates of functional and immunological remission were slightly lower, with 13% of patients failing to achieve either clinical, functional, or immunological remission. Therefore, there is an urgent need to develop new agents and treatment strategies. For example, tezepelumab, the first biological drug that targets TSLP [33], was recently approved for the treatment of severe asthma in the United States, European Union, Japan, and several other countries. We have reported the efficacy of the sequential use of these two biologics, which may help solve the clinical challenges associated with single-agent molecular-targeted therapies [34]. Various treatment strategies including stepwise management should be discussed.

The strength of this study is its focus on composite outcomes related to asthma remission, achievement rates, individual remission components, and predictors of clinical and deep remission. The limitations of this study are as follows: First, this was a retrospective analysis with a small number of patients; therefore, the possibility of an unintentional selection bias could not be completely excluded. Second, our biological selection, depending on each physician, could have affected the achievement rate of clinical and deep remission and the predictive factors. However, in the real world, attending physicians generally make a comprehensive selection of the appropriate biologics for each patient. Third, we did not record the control status of comorbidities. It cannot be denied that the control status of comorbidities, such as CRS, anxiety, and depression, may affect the achievement of clinical and deep remission. Fourth, although this study assessed the duration from the diagnosis of asthma to the initiation of biologics, the duration from the diagnosis of severe asthma to the initiation of biologics could not be evaluated because of a lack of data. Finally, the concept of clinical remission/deep remission has only recently been discussed, and the gold standard definition of clinical remission/deep remission may change in the future. The importance of the early introduction of biologics would be reinforced if there was an association between the period between the diagnosis of severe asthma and the administration of biologics and the achievement of clinical and deep remission. Further studies are required to address these issues.

## 5. Conclusions

In this study, we analyzed the achievement rate and predictive factors with the long-term use of biologics in patients with severe asthma and found that patients with early introduction of biologics have a high rate of deep remission. If we set asthma remission as our future goal, further research, including long-term prospective studies, will be needed to find an intervening strategy to overcome unmet individualized goals for each patient with severe asthma. In addition, attention should be focused on the timely introduction of disease-modifying treatments, including biologics.

## Figures and Tables

**Table 1:** Patient characteristics at baseline and 1-year follow-up.

Characteristics	All Patients (n = 54)	
	Baseline	One-Year Follow-Up
Clinical Characteristics		
Age (years)	61 (48–73)	-
Duration of asthma (years)	18 (4–31)	-
Asthma onset ≥ 18 years, n (%)	42 (77.8)	-
Sex: male, n (%)	28 (51.9)	-
Body mass index (kg/m^2^)	23.6 (21.2–26.8)	-
Obesity, n (%) (BMI ≥ 30 kg/m^2^)	20 (37.0)	
Former smoker, n (%)	22 (40.7)	-
Type 2 biomarker		
Blood eosinophils (/μL)	501 (320–938)	61 (0–219) *
FeNO, (ppb)	64 (37–123)	32 (20–60) *
Atopy	37 (68.5)	-
Total serum IgE (IU/mL)	256 (76–661)	-
Comorbidity		
Allergic rhinitis, n (%)	34 (63.0)	-
Chronic rhinosinusitis, n (%)	37 (68.5)	-
Uncontrolled features		
Exacerbation in the previous year, 0/1/≥2, n (%)	22/11/21(40.7/20.4/38.9)	45/7/2 (83.3/13.0/3.7) *
ACQ	1.6 (1.0–2.4)	0.7 (0.4–1.0) *
FEV_1_ % predicted (%)	77.0 (51.8–91.5)	90.8 (73.3–99.0) *
Treatment		
Use of ICS/LABA, n (%)	54 (100.0)	-
Use of LAMA, n (%)	33 (61.1)	-
Use of LTRA, n (%)	29 (53.7)	-
Use of maintenance OCS, n (%)	20 (37.0)	7 (13.0) *
Maintenance OCS dose † (mg/day)	6.0 (5.0–10.0)	0.0 (0.0–2.9) *
Type of biologics, Oma/Mep/Ben/Dup/Switch, n (%)	6/4/18/13/13 (11.1/7.4/33.3/24.1/24.1)	-
Number of each remission criterion achieved, 0/1/2/3/4/5, n (%)	4/14/23/11/2/0 (7.4/25.9/42.6/20.4/3.7/0.0)	0/7/10/3/17/17 (0.0/13.0/18.5/5.6/31.5/31.5) *

Data are presented as the median (interquartile range) or n (%). † Maintenance OCS dose is provided as prednisone-equivalent for patients on chronic OCS therapy. * *p* < 0.05 (baseline vs. 1-year follow-up). LAMA, long-acting muscarinic antagonist; LTRA, leukotriene receptor antagonist; OCS, oral corticosteroid; Oma, omalizumab; Mep, mepolizumab; Ben, benralizumab; Dup, dupilumab; Switch, switched to biologic from other biologics; ACQ, asthma control questionnaire.

**Table 2:** Comparison of patient characteristics between the clinical remission and non-clinical remission groups.

Characteristics	Clinical Remission (n = 37)	Non-Clinical Remission (n = 17)	*p*-Value
Clinical characteristics			
Age (years)	64 (54–73)	55 (47–73)	0.333
Duration of asthma (years)	13 (4–28)	27 (16–40)	0.006
Asthma onset ≥ 18 years, n (%)	32 (86.5)	10 (58.8)	0.035
Sex: male, n (%)	17 (46.0)	11 (64.7)	0.249
Body mass index (kg/m^2^)	23.6 (20.5–26.1)	25.0 (20.5–26.1)	0.208
Obesity	11 (29.7)	9 (52.9)	0.134
Former smoker, n (%)	10 (27.0)	12 (70.6)	0.004
Type 2 biomarker			
Blood eosinophils (/μL)	503 (372–909)	478 (216–1153)	0.635
FeNO (ppb)	67 (33–133)	56 (42–101)	0.802
Atopy	27 (73.0)	10 (58.8)	0.353
Total serum IgE (IU/mL)	347 (81–662)	199 (70–675)	0.635
Comorbidity			
Allergic rhinitis, n (%)	24 (64.9)	10 (58.8)	0.765
Chronic sinusitis, n (%)	25 (67.6)	12 (70.6)	1.000
Uncontrolled features			
Exacerbation in the previous year, 0/1/≥2, n (%)	17/8/12 (46.0/21.6/32.4)	5/3/9 (29.4/17.7/52.9)	0.140
ACQ	1.6 (1.0–2.2)	2.4 (1.2–2.4)	0.011
FEV_1_ % predicted (%)	91.5 (78.0–96.4)	71.5 (49.4–84.2)	0.0005
Treatment			
Use of ICS/LABA, n (%)	17 (100.0)	37 (100.0)	
Use of LAMA, n (%)	21 (56.8)	12 (70.6)	0.383
Use of maintenance OCS, n (%)	10 (27.0)	10 (58.8)	0.035
Maintenance OCS dose † (mg/day)	8.0 (5.0–11.3)	5.5 (5.0–10.0)	0.501
Type of biologics, Oma/Mep/Ben/Dup/Switch, n (%)	4/1/14/11/7 (10.8/2.7/37.8/29.7/18.9)	6/4/12/6/9 (16.2/10.8/32.4/16.2/24.3)	0.131

Data are presented as the median (interquartile range) or n (%). † Maintenance OCS dose is provided as prednisone-equivalent for patients on chronic OCS therapy. LAMA, long-acting muscarinic antagonist; LTRA, leukotriene receptor antagonist; OCS, oral corticosteroid; Oma, omalizumab; Mep, mepolizumab; Ben, benralizumab; Dup, dupilumab; Switch, switched to biologic from other biologics; ACQ, asthma control questionnaire.

**Table 3:** Comparison of patient characteristics between the deep remission and non-deep remission groups.

Characteristics	Deep Remission (n = 17)	Non-Deep Remission (n = 37)	*p*-Value
Clinical characteristics			
Age (years)	62 (52–72)	61 (48–73)	0.926
Duration of asthma (years)	5 (2–23)	19 (10–36)	0.006
Asthma onset ≥ 18 years, n (%)	16 (94.1)	26 (70.3)	0.078
Sex: male, n (%)	6 (35.3)	22 (59.5)	0.144
Body mass index (kg/m^2^)	23.6 (20.0–25.5)	23.6 (21.4–28.0)	0.412
Obesity	4 (23.5)	16 (43.2)	0.229
Former smoker, n (%)	5 (29.4)	17 (46.0)	0.372
Type 2 biomarker			
Blood eosinophils (/μL)	580 (430–893)	478 (314–953)	0.301
FeNO (ppb)	43 (19–96)	67 (44–143)	0.053
Atopy	9 (52.9)	28 (75.7)	0.121
Total serum IgE (IU/mL)	219 (74–659)	283 (75–675)	0.752
Comorbidity			
Allergic rhinitis, n (%)	11 (64.7)	23 (62.2)	1.000
Chronic sinusitis, n (%)	12 (70.6)	25 (67.6)	1.000
Uncontrolled features			
Exacerbation in the previous year, 0/1/≥2, n (%)	7/4/6 (41.2/23.5/35.3)	15/7/15 (40.5/18.9/40.5)	0.791
ACQ	1.6 (1.1–2.4)	1.6 (1.0–2.4)	0.501
FEV_1_ % predicted (%)	91.5 (78.0–96.4)	71.5 (49.4–84.2)	0.0005
Treatment			
Use of ICS/LABA, n (%)	17 (100.0)	37 (100.0)	
Use of LAMA, n (%)	7 (41.2)	26 (70.3)	0.070
Use of maintenance OCS, n (%)	5 (29.4)	15 (40.5)	0.549
Maintenance OCS dose † (mg/day)	10.0 (5.5–12.5)	5.0 (5.0–10.0)	0.217
Type of biologics, Oma/Mep/Ben/Dup/Switch, n (%)	0/0/6/7/4 (0.0/0.0/35.3/41.2/23.5)	6/4/12/6/9 (16.2/10.8/32.4/16.2/24.3)	0.106
Number of each remission criterion achieved	2 (1.5–3)	2 (1–2)	0.293

Data are presented as the median (interquartile range) or n (%). † Maintenance OCS dose is provided as prednisone-equivalent for patients on chronic OCS therapy. LAMA, long-acting muscarinic antagonist; LTRA, leukotriene receptor antagonist; OCS, oral corticosteroid; Oma, omalizumab; Mep, mepolizumab; Ben, benralizumab; Dup, dupilumab; Switch, switched to biologic from other biologics; ACQ, asthma control questionnaire.

**Table 4:** Predictors of clinical remission to biologics.

Predictor	OR (95% CI)	*p*-Value
Asthma duration ≤ 5 years	10.91 (1.30–91.27)	0.028
FEV_1_ ≥ 75% predicted	3.38 (1.017–11.26)	0.047

OR, odds ratio; CI, confidence interval.

**Table 5:** Predictors of deep remission to biologics.

Predictor	OR (95% CI)	*p*-Value
Asthma duration ≤ 5 years	7.38 (2.01–27.16)	0.0026
FEV_1_ ≥ 75% predicted	26.29 (3.13–220.47)	0.0026

OR, odds ratio; CI, confidence interval.

## Data Availability

The data generated and/or analyzed during the current study are included in this published article. Additional data are available from the corresponding author upon request.
